# Study on the inherent magnetism and its relationship with mechanical properties of structural round steel

**DOI:** 10.1038/s41598-022-20718-2

**Published:** 2022-09-27

**Authors:** Yang Liu, Kun Liu, Wentao Wang, Linlin Fan, Binbin Li, Tao Yang

**Affiliations:** 1 School of Urban Planning and Municipal Engineering, Xi’an Polytechnic University, Xi’an, 710048 China; 2 State Key Laboratory of Green Building in Western China, Xi’an University of Architecture and Technology, Xi’an, 710055 China; 3 Department of Civil and Environmental Engineering, University of Michigan, Ann Arbor, MI 48109 USA

**Keywords:** Structural materials, Mechanical properties, Metals and alloys

## Abstract

Inherent magnetism is an important properties of ferromagnetic materials. In this study, the internal magnetic field intensity (IMFI) and internal magneto-mechanical effect (IMME) of Q390B in a structural field were investigated to detect and verify the inherent magnetism in structural settings. In the IMFI test, the magnetic flux was used to detect the change in magnetic field to verify the existence of magnetism. In the IMME test, a novel instrument was implemented to measure the magnetic variation in the Q390B specimen without magnetic flux. Based on the low frequency cyclic (LFC) tensile loading tests, the inherent magnetism was fully described. Experimental results indicate that IMME shows great potential and more efficiency in inherent magnetism studies and can be promoted in the near future.

## Introduction

The magnetism properties of materials are widely used in various industries^1–4^. For example, magnetism is used as a nondestructive testing method for building steel structures. However, structural steel exhausts can fail during service, which may lead to disasters. Therefore, monitoring the stress of structural steel members is vital. Structural steel, a low carbon alloy steel for buildings and bridges, is a ferromagnetic material or soft magnetic material^3,5^. After smelting, welding and manufacturing, steel components can obtain a certain magnetism referred to as inherent magnetism. In recent research studies, magnetism has drawn much attention and is widely used in material nondestructive testing (NDT)^6,7^.

Based on the magnetization processes in steels, popular approaches include magnetic Barkhausen noise (MBN), magnetic hysteresis measurement (MHM), metal magnetic memory (MMM), and magnetic flux leakage (MFL) methods^4,8–10^. The inherent dependence of magnetic properties on atomic structure and microstructure leads to a certain relation between magnetic properties and mechanical stresses^11^. The magnetic variation in structural steel is sensitive to internal defects and external loads. Under the influence of mechanical stresses, the change in intrinsic magnetization is piezomagnetic for a ferromagnetic material. In 1865, Villari found that magnetism is subjected to mechanical actions such as tension or compression^12,13^. The change in the magnetism was caused by mechanical stress^14^. The results show that structural steel tension produces an increase in magnetization in weak fields and a decrease in strong fields. In fact, the stress of ferromagnetic materials is evaluated by the change in the magnetic field. The stress state of ferromagnetic materials can be assessed by the magnetic field intensity. Since then, the connection between the piezomagnetic field and applied stress has become a hot topic^6,15^.

For origins of the magneto-mechanical effect, Jiles^4,15^ presented a series of tests for the effect of uniaxial tensile stresses of up to 85 MPa on the Barkhausen activity and magnetic properties of steels. He proposed a theory of the magneto-mechanical effect model for MBN. The domain walls are unpinned by applying stress that makes the walls to move thus changing the magnetization. Dubov^16^ conducted a study of metal properties with the nondestructive testing (NDT) technique using magnetic memory as a method. Sablik et al.^17,18^ studied the biaxial stress effects on the MHM of steel with the stress field, and elucidated the variation in magnetic properties with grain size and dislocation density were considered. Bulte et al.^19^ presented a hypothesis to explain the mechanism by which externally applied stresses can affect the magnetic properties of ferromagnetic materials. According to Leng et al.^20,21^, the MMM signal response to plastic deformation of low carbon steel was explored by an experimental investigation. Wang et al.^7^ proposed a new method for estimating the location of the stress concentration and evaluating the degree of damage using a gradient curve.

Liu et al.^22^ studied the leakage magnetic field distribution on the surface of a reinforced bar in a bridge with axial tensile stress based on the magnetic memory effect of ferromagnetic materials. Guo et al.^23^ investigated the suitability of MMM technology for monitoring the damage in steel structures exposed to complex stresses in a pseudostatic test of a portal frame. Chen et al.^24^ conducted a series of static tensile tests and MMM measurements were carried out on the commonly used Q235 steel. Su et al.^25^ studied on the relationship between the strain and metal magnetic memory field of steel beams with four-point bending tests of Q235B steel I-beams. Li et al.^26^ demonstrated that data acquisition can be realized accurately using magnetic flux leakage inspection technology based on LabVIEW and that the distribution of the spectrum entropy can provide a method for monitoring crack growth through diagnosis of internal stress concentrations in materials.

In addition, Shi et al.^2,9^ promised a magnetoelastoplastic coupling model of ferromagnetic material with plastic deformation under applied stress and magnetic fields. A general nonlinear magneto-mechanical model for ferromagnetic materials was discussed under a constant weak magnetic field^2^. Moreover, Kachniarz et al.^27,28^ designed steel frame-shaped samples with both magnetizing and sensing windings coiled on their columns, and has made tentative explorations of the magnetoelastic characteristics under tensile stresses. Furthermore, Zhang et al.^29^ and Bao et al.^14^ observed that piezomagnetic signals can be detected using fluxgate magnetometers and probed the piezomagnetic behaviour of ferromagnetic steels are subjected to tensile stress. Kaleta et al.^30^ used the magnetovision as a tool for the investigation of the fatigue process of ferromagnetics materials. Weng et al.^31,32^ established an online nondestructive stress testing method for arch bridge suspenders based on the principle of magnetic coupling. In the abovementioned previous studies, the relationship between inherent magnetism and structural steel stress has rarely been mentioned. Therefore, to address this in needs, in this paper, uniaxial tensile tests were designed to establish a connection between the magnetic variation and stress.

In the current paper, the inherent magnetism of structural round steel and the relationship between inherent magnetism and stress were studied for low-alloy high-tensile structural Q390B steel. First, the experimental specimens were prepared and internal magnetic field intensity (IMFI) tests were designed. Then, an IMFI study was carried out. Furthermore, internal magneto-mechanical effect (IMME) tests were designed to discover the relationship between the internal magneto and mechanical effect under the low-frequency cyclic (LFC) tensile loading tests. Finally, the observed results were discussed in detail, and an internal magneto-mechanical effect model in relation to stress was proposed.

## Internal magnetic field intensity (IMFI) test of structural steel

### Method of IMFI tests of structural steel

In the tests, three 32 mm structural round Q390B steel specimens were prepared in advance. The internal magnetic field of the body was considered to be uniform at the cross section of the round steel. The IMFI test of structural steel was designed with room temperature without electromagnetic interference.

#### Materials

The test round steel bar specimens were cut from the same round steel to ensure the stability of the original parameters. In Table 1, material composition and mechanical properties of structural Q390B steel specimens are provided according to the Chinese Standard^33^. These specimens are low-alloy high-strength structural steels. ‘Q390’ indicates that the yield limit value is 390 MPa. ‘B’ refers to low carbon steel. The steel specimens used in the tests are homogeneous.

**Table 1:** Material composition and mechanical properties of specimens.

Type of structural steel	Chemical component (%)								Mechanical property		
	C	Si	Mn	P	S	V	Nb	Ti	R_e_ (MPa)	R_m_ (MPa)	A (%)
Q390B	0.15	0.45	1.30	0.02	0.035	0.10	0.04	0.10	400	600	19

#### Test apparatus

**Figure 1:**
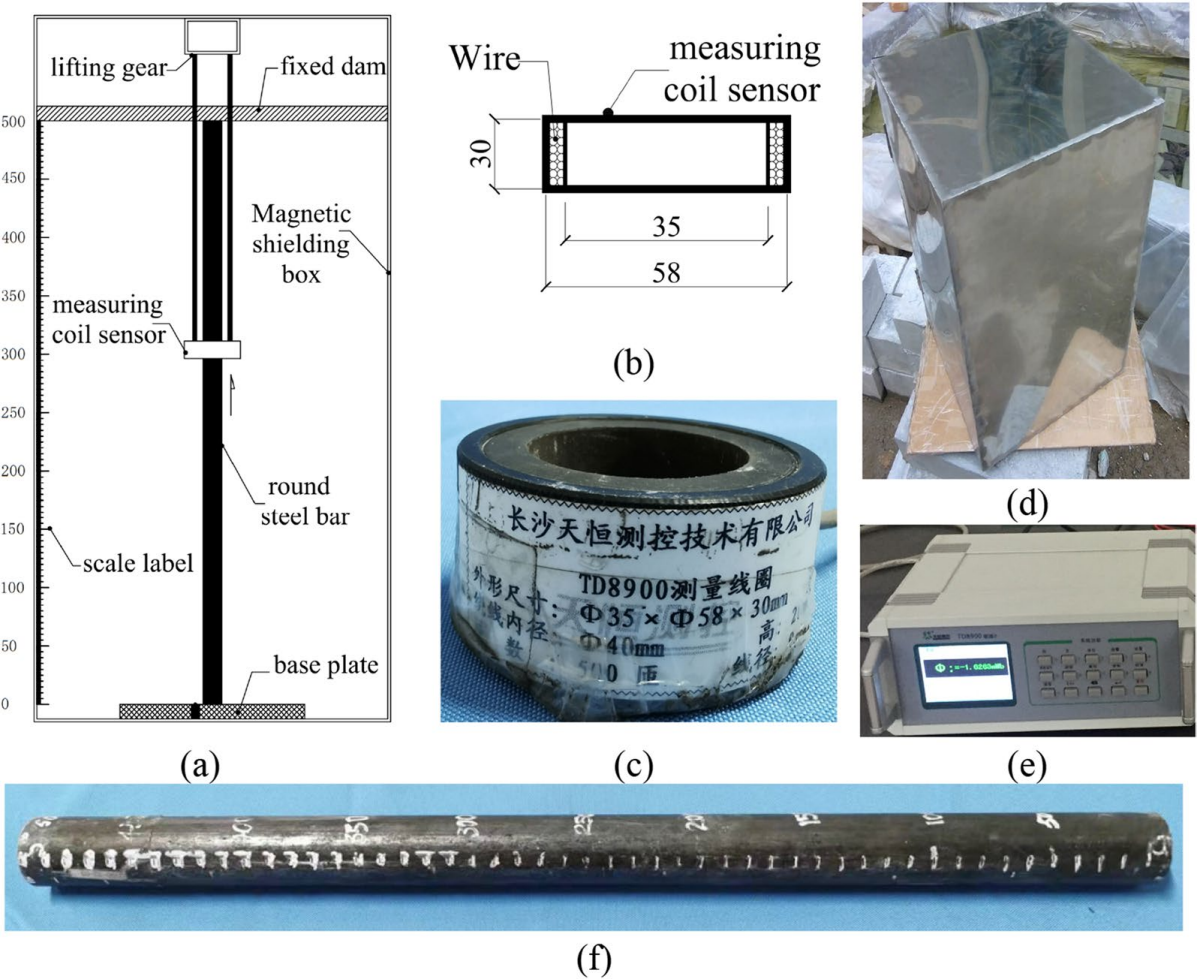
Schematic diagram of the experimental equipment. (**a**) Schematic diagram of the IMFI test; (**b**) schematic diagram of the measuring coil sensor; (**c**) measuring coil sensor; (**d**) magnetic shielding box; (**e**) TD8900 fluxmeter; (**f**) round structural steel bar specimen and scale label.

The internal magnetic field intensity (IMFI) distribution of the inherent magnetism for the round steel bar test was carried out with the experimental equipment as shown in Fig. 1a. The test device was set in the shielding box to prevent the interference caused by an external magnetic field. A schematic diagram of the IMFI test is shown in Fig. 1a.

The measuring coil sensor is a winding device as shown in Fig. 1b and c. The device is wounded 500 times by a 0.18 mm copper wire with an inner diameter of 40 mm. Its external dimensions are as follows: the inside diameter is 35 mm, the outside diameter is 58 mm, and the length is 30 mm as shown in Fig. 1b and c. The parameters for measuring the coil sensor are shown in Table 2. The size of the shielding box is 350 mm × 350 mm × 500 mm, as shown in Fig. 1d. The thickness of the shielding box is 0.5 mm with the initial permeability *μ*_0_ = 68.8 mH/m, the maximum permeability *μ*_m_ = 377.5 mH/m, the coercivity H_c_ = 0.5 A/m, and the saturation magnetic induction intensity Bs = 0.75 t. The fluxmeter, as shown in Fig. 1e, is a compact instrument, with high accuracy, automatic drift correction, and microprocessor control, to allow the operator to configure the meter for maximum resolution and accuracy. Magnetic flux is used to inversely calculate the magnetic field distribution inside the magnet. The magnetic flux value of each measuring point is measured by the fluxmeter used in the experiment. The distance spacing of the measuring points was 50 mm. As shown in Fig. 1e, the magnetic flux value was collected by a TD8900 Fluxmeter manufactured by Changsha Tianheng Measurement and Control Technology Co., LTD. The steel bar is fixed in the shielding box. In the test, the diameter of the round steel specimen is 30 mm and the length is 500 mm. The round steel bar specimen and scale label are shown in Fig. 1f.

**Table 2:** Parameters for the measuring coil sensor.

Name	Inner diameter (mm)	Outer diameter (mm)	Length (mm)	Wire diameter (mm)	Number of windings (mm)
measuring coil	35	58	30	0.16	500

### Design of the IMFI tests of structural steel

During the test, the measuring coil sensor was wrapped around the steel laterally and moved from the bottom to the top, as shown in Fig. 1a.

#### Test procedure

In the experiment, only the change in the relative magnetic flux was considered and measured at a specified point along the axial direction. There are two options to perform this experiment. One option is to first set the fluxmeter to zero, and the initial magnetic flux will be generated when the measuring coil sensor begins to move to the end because of the change in the actual magnetic field at the end of the round steel specimen. Another option is to set the fluxmeter as zero after determining the starting position of the specified measuring point. In such a case, the value of the first measuring point will be zero.

In this paper, the last option was chosen to conduct the IMFI tests of structural steel. First, the measurement sensor was moved to the specified measurement point position. Then the magnetic fluxmeter was set to zero and began to record the data of the specified point prepared. The magnetic flux values at the specified measuring points were measured along the coordinate position of the specimen. As shown in Fig. 1a, the positions of the specified measuring points were 0, 50 mm, 100 mm, 150 mm, 200 mm, 250 mm, 300 mm, 350 mm, 400 mm, 450 mm, and 500 mm, i.e., the forward method. After the measurement, the coordinate positions along the round steel are: 500 mm, 450 mm, 400 mm, 350 mm, 300 mm, 250 mm, 200 mm, 150 mm, 100 mm, 50 mm, and 0 mm, i.e., the reverse method. After the bidirectional measurement, the test was finished. Therefore, the measurement error was reduced by taking the mean value of the bidirectional measurement.

The tests were designed and conducted to obtain the internal magnetic field intensity distribution of the inherent magnetism for round steel specimens with the condition of no load and no external magnetic field in the at room temperature environment. According to the change in magnetic flux inside the round steel body, the distribution of magnetic field intensity B inside the round steel body along the length can be calculated by the Eq. (2) changed from Eq. (1).

1 $$\Phi = n \cdot B \cdot S$$

2 $$B = \frac{\Phi }{n \cdot S}$$

where $\Phi$ is the magnetic flux; *n* is the number of windings for the measuring coil sensor; *B* is the magnetic field intensity inside the round steel body; and *S* is the area of the magnetic field intensity inside the round steel body.

### Results of the IMFI test of structural steel

**Figure 2:**
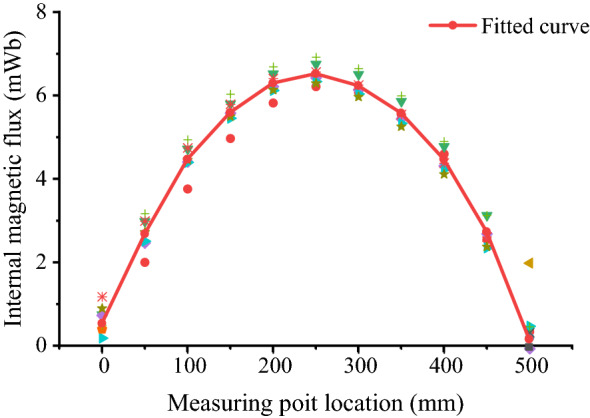
The internal magnetic flux field distribution curve of the inherent magnetism for the round Q390B steel bar.

The experimental tests were conducted at a temperature of 20 ± 3 °C. In the IMFI test, three 32 mm specimens of round steel bars were tested, and experimental data of the testing points were recorded by fluxmeter software on a computer. In the tests, the forward and reverse contrast method was used to eliminate the error. Through the test, it was found that the existing magnetism in the round steel was not affected by the external environment. The internal magnetic flux field distribution of the structural round steel bar specimen at the cross section of the round Q390B steel is shown in Fig. 2.

## Internal magneto-mechanical effect (IMME) test of structural steel

### Method of the IMME test of structural steel

In the IMME tests, the round steel member was taken as the research object. The relationship between the internal magnetic field intensity and the stress for the round steel bar was very important for detecting the stress and strain of round steel under loading. In the tensile test, the position of the section neck was difficult to determine after the material entered the plastic deformation stage. This was related to the composition of the metal materials and casting defects. Therefore, the relationship between magnetic variation and stress was designed within a certain tensile stress range. The change in the magnetic field intensity in the round steel specimen causes a change in the electric current in the measuring coil sensor. The value of the change can reflect the changing law of the magnetic field in a round steel member.

The stress of the round steel bar was determined by the uniaxial tensile test. Under the action of stress, the round steel will cause strain along the length and the direction of the cross section. At the same time, the strain along the length and the direction of the cross section will cause a change in the magnetic field intensity inside the round steel. Therefore, the relationship between stress and magnetic field intensity can be obtained through data processing and analysis. Based on this method, a relationship test between magnetic field intensity variation and stress was designed.

### Design of the relationship test between magnetic variation and strain

#### Experimental instrument

**Figure 3:**
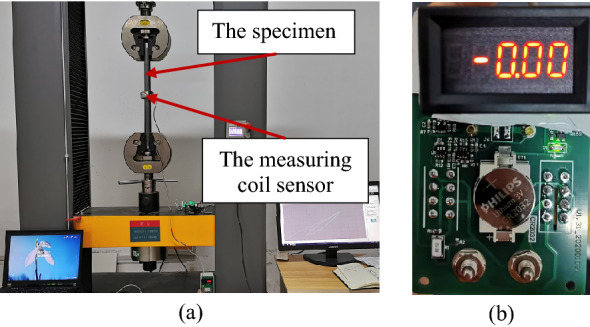
Schematic diagram of IMME test equipment. (**a**) Electrohydraulic servo testing machine and testing schematic diagram; (**b**) the current intensity measuring system.

The loading and unloading of the tensile test were completed by the universal material mechanics testing machine as shown in the Fig. 3a shown. This instrument was a microcomputer-controlled motor universal testing machine WDW-300 made by Jinan EAST Testing Instrument Co., LTD., as shown in Fig. 2a. The performance parameters are as follows: power: 1.5 kW; voltage: 220 V; accuracy:  ± 0.5%; speed range: 0.05–500 mm/min; maximum load: 300 kN; stretch space: 650 mm. Moreover, the measuring coil sensor was still wrapped in the lateral direction of the round steel bar, as shown in the middle. The measuring coil sensor was a winding device as shown in Fig. 1b an c, and the detailed parameters are listed in Table 2. In this study, the microcurrent measuring instrument was improved to measure the internal magnetic field intensity of the 32 mm structural round Q390B steel specimens. The current intensity measuring system was used to measure the change in the internal magnetic field intensity during axial loading according to the designed loading scheme. The current intensity measuring system has three measurement switching gears, i.e., nA, μA, and mA, to choose from as shown in the Fig. 3b.

#### Test procedure

The test was conducted under the conditions of constant temperature and humidity. The test temperature was kept at 20 ± 3 °C and the humidity was between 25 and 28%. During the loading process, the interference of the environmental magnetic field was ignored. According to the designed tensile strength of 335 MPa (Code for design of steel structures, China), the relationship between the internal magnetic field intensity and the stress for round steel bars was just studied within the elastic range. Finally, the loading scheme is shown in Table 1. The loading speed was 10 MPa/s and the maximum load was selected as 330 MPa. The proportional limit stress σ_P_ was determined by tensile testing. The load target was 60 kN, the real stress is 75 MPa, and the specimens were in the elastic phase.

**Figure 4:**
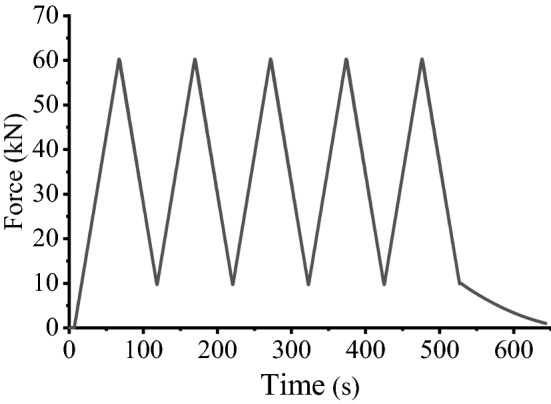
Test loading force and time t curve.

The universal material mechanics testing machine was equipped with a nonconductive fixture. The load path was selected within the elastic range for the structural round Q390B steel specimens. The preloading rate was determined to be 5 mm/min, and the preload was 1 kN. After reaching the target value of 1 kN, the machine started loading at a rate of 1 kN/s. During the test, the value of magnetic flux at the middle point of the specimen was measured and recorded synchronously. The test was repeated 5 times, each load ranging from 0 to 60 kN. The whole process was as follows: First, the test specimen with measuring coil was fixed, and the test fixture was tightened. Second, the motor universal testing machine was stretched to the specified value of 60 kN at the rate shown in Table 3, which was maintained for 5 s. Finally, after the holding time was reached, unloading to 10 kN was performed at the same rate, and then this process was repeated 5 times, as shown in Fig. 4.

**Table 3:** Loading system table for Q390B.

Type of structural steel	Diameter Φ (mm)	Length (mm)	The maximum load (MPa)	Loading speed (kN/s)	Load target (kN)
Q390B	32	500	330	1	60

In the study, the existing magnetic variation characteristics at the mid-span point of the round steel specimen were selected. A special bandage was used to hold the measuring coil at the centre of the round steel specimen. In this way, the measuring coil was kept in the middle of the specimen during the tensile test.

### Results of the relationship test between magnetic variation and strain

**Figure 5:**
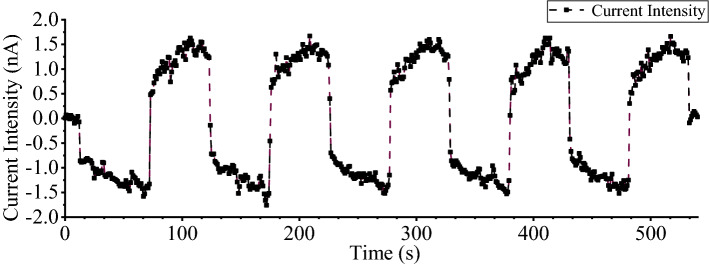
Current intensity and time curve.

Through the experiment, we can conclude that the measuring coil is produced by the induction current in the measurement, which can be given the strength value of the field by collecting, integrating and processing the current size. The change in IMME at the midpoint of the round steel specimen under LFC tensile loading and unloading. The experimental data of the testing points were recorded by the current intensity measuring system. According to the testing results on the universal tester above, the current intensity change curve of the time-varying process in the test is obtained, as shown in Fig. 5. Under the action of axial tension, the stress produced by the steel axial force deforms the whole steel body, which reflects the strain, longitudinal tension and transverse contraction.

## Discussion

### Internal magnetic field intensity (IMFI) of inherent magnetism

**Figure 6:**
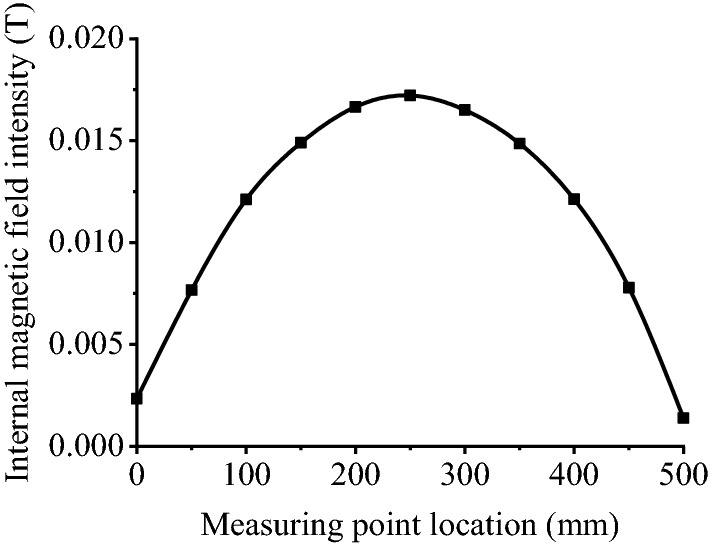
The internal magnetic field intensity curve.

The internal magnetic field distribution curve of the inherent magnetism for the round Q390B steel bar was converted at the cross section of the round steel, as shown in Fig. 2. The fitted curve is also shown in Fig. 2. The measurement results and the fitted curve can be used to obtain the value of the magnetic field intensity by transformation of Eq. (2). The internal magnetic field intensity curve is shown in Fig. 6.

The x-axis is the position of the measuring point along the length of the round steel, and the y-axis is the magnetic field intensity. The intensity of the magnetic field increases from one end to the other, reaches its maximum at the midpoint, and decreases afterwards. It can be seen from the results that the magnetic field intensity is the maximum at the middle of the round steel. The magnetic field intensity is 0.0172 T at the midpoint of the specimen. The fitting results show that the intensity of the magnetic field distribution is parabolic along the length direction. According to the test results shown in Fig. 6, the following equation was obtained from the fitting of a polynomial:

3 $$B = - 2.446 \times x^{2} + 1.215 \times x + 0.002\quad ({\text{R}}^{2} = 0.998)$$

Here, x is the distance of the specimen with dimensions in millimetres (mm). For the round steel specimen, the intensity distribution of the magnetic field is related to the size of the round steel. Furthermore, the production and casting of the structural steel was analysed after a series of technological processes, including argon blowing in the converter, ladle bottom, wire feeding, refining, slab continuous casting, heating, descaling, cooling, etc. During smelting and casting, the structural steel was in the form of sheet steel, and section steel or bar steel served as the raw material of the steel structure. In this process, the refined molten steel crystallizes into a slab through the slab continuous casting process at a reduced temperature. During high-temperature continuous casting, heating and cooling, iron atoms and carbon atoms are dissolved. Temperature changes in smelting and casting promote the dissolution of carbon atoms in the lattice and the mutual transformation of austenite and martensite. All procedures mentioned above affect the magnetic properties of the structural steel.

### Internal magneto-mechanical effect (IMME)

**Figure 7:**
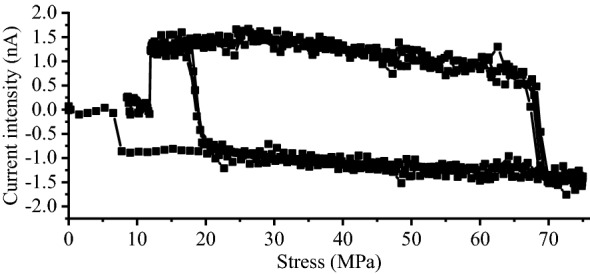
Current intensity and force curve.

**Figure 8:**
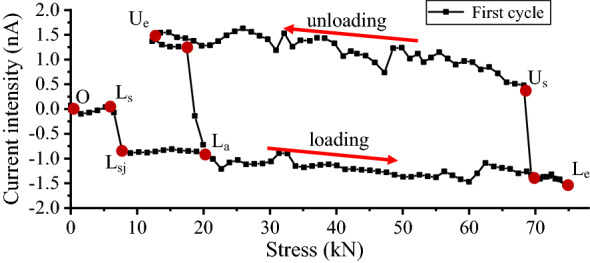
Current intensity and force curve of the first LFC test.

For the specimen, the magnetic field intensity of inherent magnetism is 0.0172 T at the midpoint. In the elastic stage, when the load is loaded to 60 kN, the actual axial stress is 75 MPa. At this moment, the relation diagram of the current intensity variation and force after five cycles of LFC tensile loading and unloading are shown in Fig. 7. The first tensile loading and unloading cycle in the test is shown in Fig. 8. The first main loading stage is from point Ls to Le. As the load increases, the current generated by the magnetic field changes increases. Continuous output current from the measuring coil sensor can be seen from the figure. Point O to Le is a preloading stage. The curve jump point Lsj occurs at the beginning of the loading. The value of La is 0.68 nA with a force of 16 kN. Subsequently, the current intensity change increases to − 1.58 nA with a force of 60 kN. Point Le is a turning point from the loading and unloading transition. The results of the current intensity jump from point Le to Us. In the unloading stage, the curve is from point Le to Us to Ue. The current intensity change increases to 1.55 nA with a force of 55 kN. The value of Us is 0.50 nA with a force of 10 kN. The second cycle began loading from point Ue to La to Le. The curve jump point occurs during the loading and unloading transition or the unloading and loading transition.

**Figure 9:**
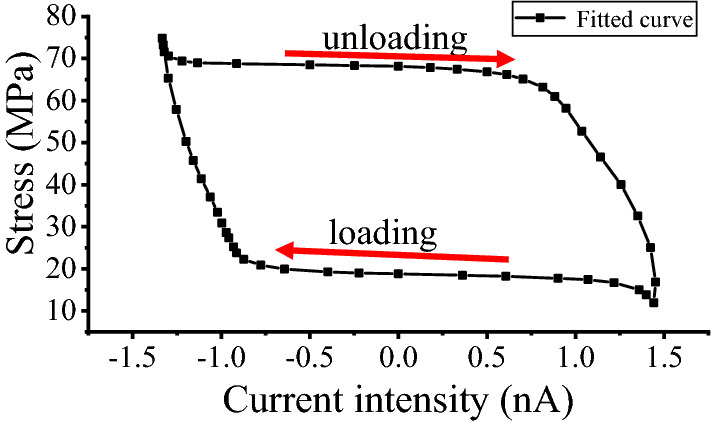
Current intensity and fitted stress curve of the LFC tests.

By changing the coordinate system, the relation of the current intensity and fitted curve stress is shown in Fig. 9. When the force was loaded to 60 kN, the actual axial stress of the cross-sectional stress of the structural round steel specimen was 75 MPa. The internal magnetomechanical effect model was derived as follows.

In the loading stage, the cross-section stress $\sigma_{loading}$ is fitted as Eq. (4) by the first-order decay exponential function for the midpoint of the specimen.

4 $$\sigma_{loading} = 16.457 + 0.212 \times e^{{ - \frac{I}{1.369}}} \quad ({\text{R}}^{2} = 0.9929)$$

In the unloading stage, the cross-section stress $\sigma_{unloading}$ is fitted as Eq. (5) by the first-order decay exponential function for the midpoint of the specimen.

5 $$\sigma_{unloading} = 70.217 - 0.748 \times e^{{\frac{I}{1.956}}} \quad ({\text{R}}^{2} = 0.9854)$$

For the loading stage, the value $\Delta \sigma_{loading}$ of different *t*_2_ and *t*_1_ values can be derived from Eq. (4).

6 $$\Delta \sigma_{loading} = 0.102 \times e^{{I_{2} }} (1 - e^{{(I_{1} - I_{2} )}} )$$

Here, I_t2_ and I_t1_ are the values of the current intensity at the start and end, respectively; e is a constant, also the base of the natural logarithm; and $\Delta \sigma_{loading}$ is the value of the stress difference between I_2_ and I_1_. The internal magnetomechanical effect model in Eqs. (4) and (5) shows that the relationship between the current intensity and force is stable under the uniaxial stress of round steel in the elastic range. The change between I_2_ and I_1_ for the 32 mm structural round Q390B steel specimens can be calculated by the fitted exponential function Eq. (6). The formulas will also be used in detecting and monitoring the stress changes for steel structural members in civil or mechanical industries.

For metallic materials, especially polycrystalline metallic materials, the changes in internal crystal structure are very complex under the action of external forces. For structural steels, the magnetic properties are even more complex for steel members under complicated stress. The study of complex magnetism involves many subjects, such as metal material science, microstructure characterization, quantum mechanics, and micromechanics. For traditional mechanics, the mechanical properties of structural steels cannot be explained clearly. In the tests, the relationship between the current intensity and force is stable under the uniaxial stress of round steel in the elastic range. The relationship between stress and magnetic properties can reflect the mechanical relationship. The relationship can be used to predict the real-time stress condition of steel structural members. By analysing the results of the two experiments, the relationship between the change in stress at the middle point of the round steel and the change in magnetic field intensity is obtained. Finally, it should be noted that the relationship between the internal magnetic field intensity and the stress of the round steel bar is very important for detecting the stress and strain of round steel under loading.

## Conclusions

In this paper, an experimental approach was taken to determine the connection between the internal magnetic field intensity and the stress for round steel bars in the elastic range of the material. The results of the study aim to propose a novel way to measure stress changes, which will also be used in detecting and monitoring stress changes for steel structural members in civil or mechanical industries.

1. In IMFI tests, the internal magnetic field intensity is not evenly distributed along the length, showing a symmetrical distribution. The maximum value appears at the middle point of the round steel bar. The fitting results show that the intensity of the magnetic field distribution is parabolic along the length direction, as shown in Eq. (3).
2. In IMME uniaxial tensile tests, the relationship test between the magnetic field intensity variation and stress of the existing magnetism was studied in the loading and unloading phases. The internal magnetomechanical effect model shown in Eqs. (4) and (5), demonstrates that the relationship between the current intensity and force is stable under the uniaxial stress of round steel in the elastic range.

There was a jump point for the current intensity value that occurs during the stage of the loading and unloading transition or the unloading and loading transition in the elastic stage. The internal magnetic domain of structural steel is stable, showing the characteristics of the magnetic effect under a low cyclic reciprocating load. It can be concluded that the variation in the magnetic field in round steel is regular under tension. For the 32 mm structural round Q390B steel specimens, the model changes between I_2_ and I_1_ can be calculated by the fitted exponential function, Eq. (6). Future work will focus on studies of the relationship between the stress and strain of structural steel and the magnetism of the microstructure in thermodynamics and atomic mechanics.

## Data Availability

All data generated or analyzed in the study are included within the paper.
